# Applied Dynamic System Theory for Coordination Assessment of Whole-Body Center of Mass During Different Countermovements

**DOI:** 10.3390/s26030957

**Published:** 2026-02-02

**Authors:** Carlos Rodrigues, Miguel Velhote Correia, João M. C. S. Abrantes, Marco Aurélio Benedetti Rodrigues, Jurandir Nadal

**Affiliations:** 1 Faculty of Engineering, University of Porto, 4200-465 Porto, Portugal; 2 Centre of Biomedical Engineering Research, Institute for Systems and Computer Engineering, Technology and Science, 4200-465 Porto, Portugal; 3 Centre for Research in Applied Communication, Culture and New Technologies (CICANT), Lusófona University, 1749-024 Lisbon, Portugal; 4 Department of Electronics and Systems, Federal University of Pernambuco, Recife 50670-901, Brazil; 5 Biomedical Engineering Program, COPPE/Federal University of Rio de Janeiro, Rio de Janeiro 21941-914, Brazil

**Keywords:** dynamical system, image system, force platform, COM, coordination

## Abstract

This study applies phase plane analysis of medio-lateral, anteroposterior, and vertical directions for the coordination assessment of whole-body (WB) center of mass (COM) movement during the impulse phase of a standard maximum vertical jump (MVJ) with long, short, and no countermovement (CM). A video system and force platform were used, with the amplitudes of WB COM excursion obtained from image-based motion capture at each anatomical direction, and the 2D and 3D mean radial distance were compared under long, short, and no CM conditions. The estimate of the population mean length was used as a measure of distribution concentration, and the Rayleigh statistical test for circular data was applied with the sample distribution critical value. Watson’s U^2^ goodness-of-fit test for the von Mises distribution was used with the mean direction and concentration factor. The applied metrics led to the detection of shared and specific features in the global and phase plane analysis of WB COM movement coordination in the medio-lateral, anteroposterior, and vertical directions during long, short, and no CM conditions in relation to MVJ performance assessed from ground reaction force (GRF) through the force platform. Thus, long, short, and no CM impulses share lower amplitudes of WB COM excursion in the medio-lateral direction and mean radial distance to its mean, whereas the anteroposterior and vertical excursion of WB COM, along with the 2D transversal and 3D spatial length of the WB COM path, present as potential predictors of MVJ performance, with distinct behavior in long CM compared to short and no CM. Additionally, the applied workflow on generalized phase plane analysis led to the detection, through complementary metrics, of the anatomical WB COM movement directions with higher coordination based on phase concentration tests at 5% significance, in line with MVJ performance under different CM conditions.

## 1. Introduction

Throughout the human life cycle, from birth to young age and adulthood, bipedalism plays a determinant role in the transition from crawling to walking, running, and jumping, providing advantages in posture, dexterity, and performance at ontogenetic and phylogenetic levels [1,2]. As a contact control task, human gait stability and progression critically depend on the precise control of the resultant ground reaction force (GRF) acting at the center of pressure (COP), which, along with the force of gravity acting on the whole-body (WB) center of mass (COM), is responsible for COM movement [3,4].

Biped posture and gait depend on internal muscle action to oppose gravity and produce the necessary forces responsible for the gait GRF [5]. Despite significant research effort focused on isolated isometric, concentric, and eccentric actions, the natural form of muscle action frequently involves the stretch-shortening cycle (SSC) for efficient submaximal and powerful maximal action, particularly in the lower limbs during walking, running, and jumping [6,7,8]. Although muscle SSC can be observed during walking and running, its higher expression and assessment occur during the standard maximum vertical jump (MVJ) with WB weight applied during the countermovement jump (CMJ) for long countermovement (CM) and SSC, and the drop jump from a 40 cm step (DJ) for short CM and SSC comparison with the squat jump (SJ), which involves no CM or SSC [9,10]. Despite the primary research focus on WB movement for long, short, and no CM assessment during the standard MVJ is directed toward the vertical direction, both COP and COM also move in the transverse plane.

Regardless of some studies on COP transversal excursion for dynamic postural stability assessment [11] and during ground contact in the MVJ impulsion phase [12], the majority of MVJ studies are dedicated to the COM vertical movement of the WB [13,14,15,16,17], with no studies on WB COM movement in the transversal plane, despite its relevance for stability analysis [18] during the impulse phase under each CM condition, as well as its contribution to MVJ performance [19,20,21]. For this reason, after comparing the amplitudes of COM excursion in the medio-lateral, anteroposterior, and vertical directions, mean radial distance and length of the transversal and spatial path from COM excursion during the MVJ impulse phase, we applied generalized phase plane analysis to WB COM movement in each anatomical direction, searching for hints at a low-dimensional level [22,23] of neuromuscular control assessment under each CM condition.

Developed metrics based on WB COM displacement direction provide a novel and informative framework for characterizing movement coordination during the impulse phase of vertical jumping. Proposed coordination metrics based on kinematic phase relationships may be applied to performance evaluation and technique optimization in athletic training, where differences in coordination strategies across countermovement conditions can inform individualized training interventions. In addition, these metrics have potential applications in injury risk assessment and rehabilitation monitoring, as altered coordination patterns of WB COM may reflect neuromuscular control deficits or compensatory strategies following injury.

## 2. Materials and Methods

### 2.1. Study Design

The study was designed to assess WB COM coordination under long, short, and no countermovement conditions (CMJ, DJ, and SJ), extending COM analysis beyond the vertical direction to the medio-lateral and anteroposterior planes, and expanding global metrics analysis with generalized phase space analysis. For this purpose, the study relies on kinematic and kinetic outcomes from anatomical joint reflective markers and synchronized ground reaction forces from a force platform used for phase segmentation. Each subject performs a total of three repetitions of each MVJ type, and within-subject repeated measures were applied for the minimization of inter-individual variability.

### 2.2. Participants

Due to the interest in the assessment of untrained subjects with potential for training, a total of 54 trials were acquired during 3 CMJ, 3 DJ, and 3 SJ repetitions performed by a sample of six sports students without previous injury or specific training, with an average age of 21.5 ± 1.4 years, mass of 76.7 ± 9.3 kg, and height of 1.79 ± 0.06 m.

### 2.3. Assessments

**Figure 1:**
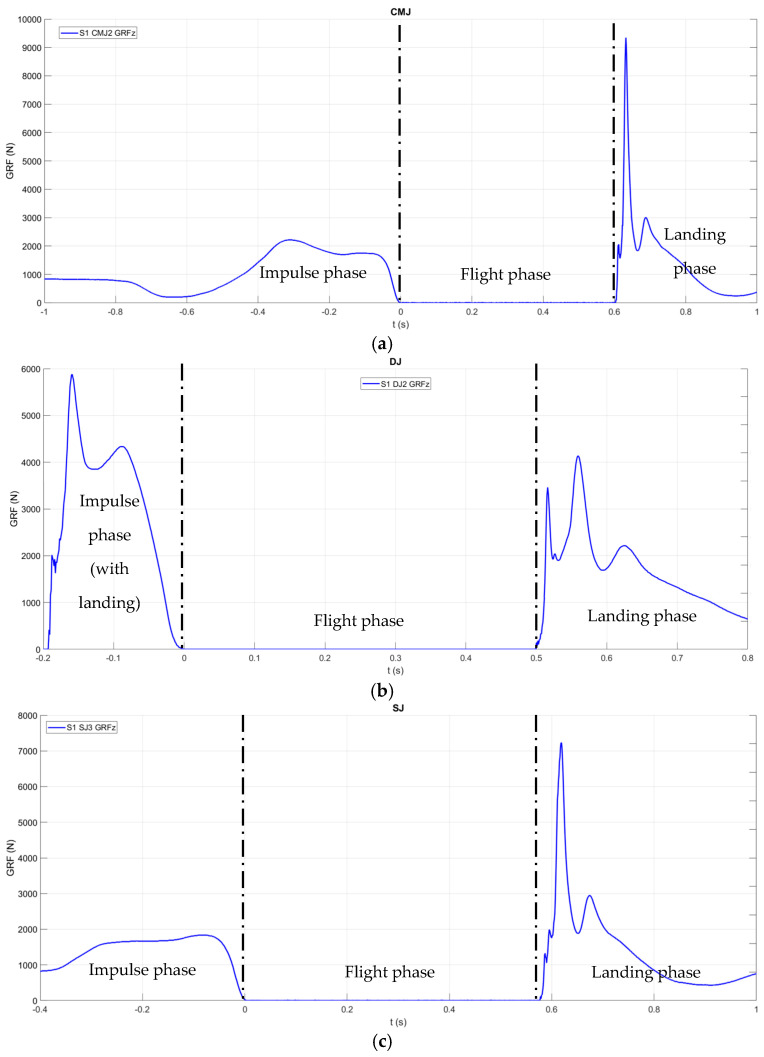
Vertical ground reaction force component (GRFz) time profiles during the impulse, flight, and landing phases of CMJ (**a**), DJ (**b**), and SJ (**c**) trials performed by subject S1.

During trial tests, ground reaction forces (GRFs) were acquired with the 3D multi-axis strain gauge force platform AMTI BP2416-4000CE (Advanced Mechanical Technology Inc., Watertown, MA, USA) at 1000 Hz coupled with a Mini Amp MSA-6 amplifier for impulse (I), flight (F), and landing (La) phase detection, as shown in Figure 1, with WB weight Fg for CMJ (I: GRFz<Fg, F: GRFz=0, La: GRFz>0), DJ (I: GRFz>0, F: GRFz=0, La: GRFz>0), and SJ (I: GRFz>Fg, F: GRFz=0, La: GRFz>0) trials, with a golf ball impact used for synchronization with the imaging system.

According to defined criteria for impulse, flight, and landing phases, CMJ, DJ, and SJ were time-segmented as shown in Figure 1, with focus on the impulse phase as the determinant of the flight phase responsible for maximum vertical jump height of the WB COM.

WB COM Cartesian coordinates (x, y, z) were obtained using the Dempster [24] modified model of 14 segments with image-based Simi Motion 6.1 (Simi Reality Motion Systems GmbH, Unterschleissheim, Germany) and image acquisition with a stereoscopic camera at a 4 m distance perpendicular to the sagittal plane from noninvasive passive reflective markers placed on the skin surface of externally palped anatomical joints on the left and right shoulder at the acromion, the left and right anterior superior iliac spines, the left and right knees at the femoral epicondyles, the left and right ankles at the lateral malleolus, the left and right heels at the calcaneus, and the heads of the left and right 2nd metatarsals. The workspace calibration of the imaging system was performed using a plane structure of known dimensions positioned at the limits of the working volume, and stereoscopic vision was implemented with a pair of JVC GR-VL9800 cameras (JVCKENWOOD Corporation, Yokohama, Kanagawa, Japan) operating at 100 Hz in anterior and posterior positions to the coronal plane, pointed at the subject in a plane parallel to the sagittal plane at a 4 m distance. Tracking of the markers during human movement was performed with Simi Motion 6.1 and direct linear transformation (DLT) was executed to obtain 3D coordinates from the 2D camera image, with digital filtering of raw data using a second-order Butterworth low-pass filter of 12 Hz applied forward and backward to eliminate phase shift.

The dynamic signal from the ground reaction force was resampled to match the 100 Hz kinematic frequency, whereas the 12 Hz low-pass filter of the kinematic signal balanced the elimination of digitalization artifacts while maintaining the larger part of the signal energy. Finally, the interpolation of the kinematic signals was performed using quintic splines prior to the derivative calculations to obtain COM velocities and accelerations.

**Figure 2:**
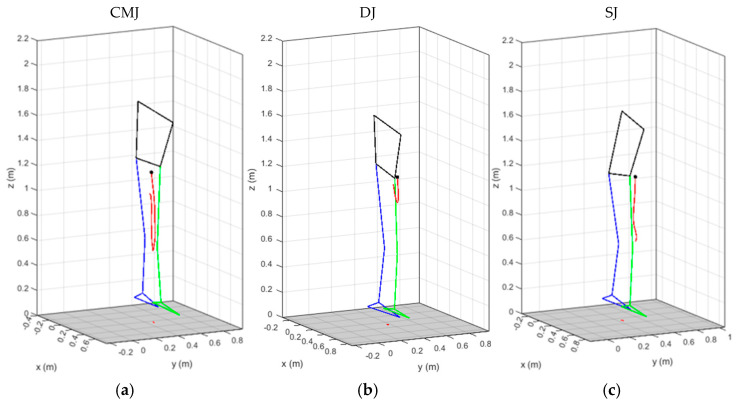
Stick-figure and WB COM trajectory during the impulse phase on CMJ (**a**), DJ, (**b**) and SJ (**c**) trials performed by subject S1, with the black circle corresponding to the take-off. ("blue: left leg; green: right leg; black: torso; red: WB COM trajectory").

**Figure 3:**
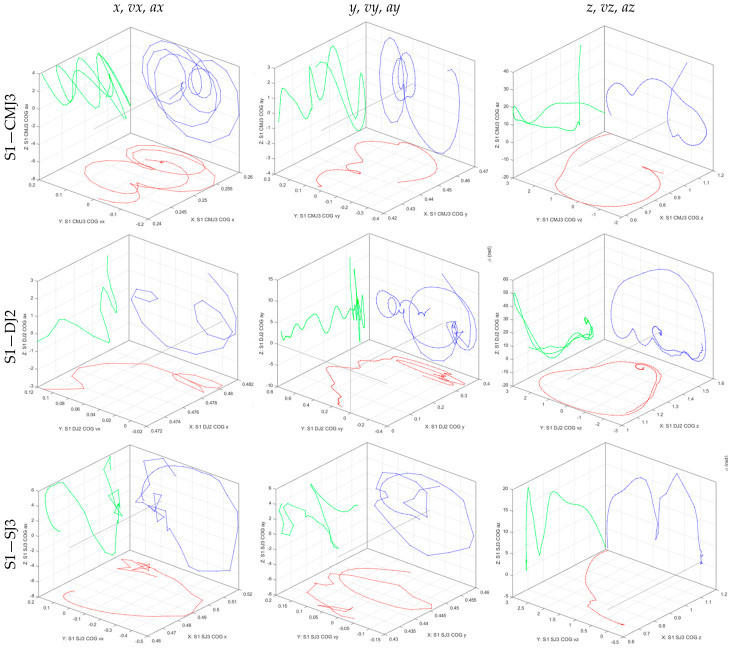
Medio-lateral (*x*, *vx*, *ax*), anteroposterior (*y*, *vy*, *ay*), and vertical (*z*, *vz*, *az*) phase planes of WB COM trajectory during the impulse phase of the CMJ, DJ, and SJ trials performed by subject S1. (“red: displacement-velocity; green: displacement-acceleration; blue: velocity-acceleration”).

Cartesian coordinates from the WB COM were exported from Simi Motion and the amplitudes (*A*) of the medio-lateral (*x*), anteroposterior (*y*), and vertical (*z*) displacements, mean radial distance (*R*), 2D (*L*) and 3D (*l*) lengths of the path during COM excursion in the impulse phase, as presented in Figure 2 for the CMJ (a), DJ (b), and SJ (c) trials, were computed using Equations (1)–(4). Phase planes were plotted using MATLAB R2017a (MathWorks Inc., Natick, MA, USA) for the medio-lateral *x*, anteroposterior *y*, and vertical *z* WB COM displacements, velocities, and accelerations, as presented in Figure 3 for the CMJ, DJ, and SJ trials performed on subject S1, with corresponding phase plane analysis during the impulse phase for each subject’s best trial of CMJ, DJ, and SJ based on MVJ flight time.

(1) $$A_{q}=\left|max\left\{{COM}_{q}^{i}\right\}-min\left\{{COM}_{q}^{i}\right\}\right|,\;q\in \left\{x,y,z\right\}$$

(2) $$R=\frac{1}{n}\sum_{i=1}^{n}\sqrt{{\left({COM}_{x}^{i}-{COM}_{avgx}\right)}^{2}+{\left({COM}_{y}^{i}-{COM}_{avgy}\right)}^{2}}$$

(3) $$L=\sum_{i=2}^{n}\sqrt{{\left({COM}_{x}^{i}-{COM}_{x}^{i-1}\right)}^{2}+{\left({COM}_{y}^{i}-{COM}_{y}^{i-1}\right)}^{2}}$$

(4) $$l=\sum_{i=2}^{n}\sqrt{{\left({COM}_{x}^{i}-{COM}_{x}^{i-1}\right)}^{2}+{\left({COM}_{y}^{i}-{COM}_{y}^{i-1}\right)}^{2}+{\left({COM}_{z}^{i}-{COM}_{z}^{i-1}\right)}^{2}}$$

Whereas CMJ impulse phase starts with the subject at orthostatic position and long countermovement with downward movement reverse to upward at 90° of knee flexion Figure 2a, DJ initiates at downward movement from a 40 cm step with the impulse phase start at landing ground contact and short countermovement with im-mediate reverse of downward to upward movement Figure 2b, SJ impulse phase starting from squat position at 90° of knee flexion with upward movement and no CM, Figure 2c, with corresponding long, short and no countermovement on CMJ, DJ and SJ as in Table 1.

WB COM displacements (q), velocities ($\dot{q}$), and accelerations ($\ddot{q}$) in the medio-lateral (*x*, *vx*, *ax*), anteroposterior (*y*, *v_y_*, *a_y_*), and vertical (*z*, *v_z_*, *a_z_*) directions, as presented in Figure 3 for the trials performed by subject S1, were normalized [25] at CMJ, DJ, and SJ using Equation (5), with i=1,…,N representing the samples at 100 Hz during impulse phases:

(5) $${\tilde{q}}_{i}=\frac{q_{i}-\left[min\left(q\right)+max\left(q\right)\right]/2}{\left[max\left(q\right)-min\left(q\right)\right]/2};{\tilde{\dot{q}}}_{i}=\frac{{\dot{q}}_{i}-\left[min\left(\dot{q}\right)+max\left(\dot{q}\right)\right]/2}{\left[max\left(\dot{q}\right)-min\left(\dot{q}\right)\right]/2};{\tilde{\ddot{q}}}_{i}=\frac{{\ddot{q}}_{i}-\left[min\left(\ddot{q}\right)+max\left(\ddot{q}\right)\right]/2}{\left[max\left(\ddot{q}\right)-min\left(\ddot{q}\right)\right]/2}$$

with previously computed $min\left(q\right)=min\left(q_{i}\right)$, $max\left(q\right)=max\left(q_{i}\right)$, $min\left(\dot{q}\right)=min\left({\dot{q}}_{i}\right)$, $max\left(\dot{q}\right)=max\left({\dot{q}}_{i}\right)$, $min\left(\ddot{q}\right)=min\left({\ddot{q}}_{i}\right)$, and $max\left(\ddot{q}\right)=max\left({\ddot{q}}_{i}\right)$ for i=1,⋯,N.

Phase space analysis was applied by generalizing the classic phase plane in Equation (6) for the medio-lateral (*x*, *vx*, *ax*), anteroposterior (*y*, *vy*, *ay*), and vertical (*z*, *vz*, *az*) directions.

(6) $$\phi_{q\dot{q}}=\tan^{-1}\left(\tilde{\dot{q}}/\tilde{q}\right);\phi_{\dot{q}\ddot{q}}=\tan^{-1}\left(\tilde{\ddot{q}}/\tilde{\dot{q}}\right);\;\;\phi_{q\ddot{q}}=\tan^{-1}\left(\tilde{\ddot{q}}/\tilde{q}\right)$$

Mean phase directions $\bar{\phi }$ were computed from Cartesian coordinates in Equation (7) and mean resultant length $\bar{r}$ with circular standard deviation *σ* from Equation (8) [26,27].

(7) $$\bar{c}=\frac{1}{n}\sum_{i=1}^{n}\cos\;\phi_{i};\bar{s}=\frac{1}{n}\sum_{i=1}^{n}\sin\;\phi_{i};\;\;\bar{\phi }=\left\{\begin{array}{c} \tan^{-1}\left(\bar{s}/\bar{c}\right)\;\;\;\;\;\;\;\;\;\;\;\;\;\;\;\;\;\;\;\;\;,\bar{c}\geq 0\\ \tan^{-1}\left(\bar{s}/\bar{c}\right)+\pi \mathrm{sig}\left(\bar{s}\right),\bar{c}<0\; \end{array}\right.$$

(8) $$\bar{r}=\sqrt{{\bar{c}}^{2}+{\bar{s}}^{2}};\;\;\;\;\;\;\;\sigma =\sqrt{-2\ln\bar{r}}$$

The amplitudes *A* of WB COM excursion, mean radial distance *R*, and 2D and 3D length *L* and *l* were then compared at each anatomical direction, along with several phase plane metrics such as the mean radial distance $\bar{r}$ based on the mean direction $\bar{\phi }$ for the generalized phase planes. The estimate $\bar{r}$ of the population mean length *ρ* was used as a measure of distribution concentration, with a null value associated with uniform distribution at the unit circle and a unit value associated with maximum concentration for a specific direction. The Rayleigh statistical test for circular data was applied using the sample distribution critical value presented in Equation (9), and Watson’s *U*^2^ goodness of fit test for the von Mises distribution was used with the mean direction and concentration factor presented in Equation (10) and $V_{i}=F\left(\phi_{i}\right)$ being the theoretical distribution of the sorted values $\phi_{i}$ according to [26,27].

(9) $$z=n\;{\bar{r}}^{2};P\left(z\geq k\right)=e^{\sqrt{1+4n+4\left(n^{2}-nk\right)}-\left(1+2n\right)}$$

(10) $$U_{n}^{2}=\sum_{i=1}^{n}V_{i}^{2}-\sum_{i=1}^{n}\frac{\left(2i-1\right)V_{i}}{n}+n\left[\frac{1}{3}-{\left(\bar{V}-\frac{1}{2}\right)}^{2}\right]$$

The mean radial distance $\bar{r}$ is a metric of the phase concentration $\bar{\phi }$, varying from the null value $\bar{r}=0$ at uniform distribution associated with lower coordination to $\bar{r}=1$ higher phase concentration for maximum variable coordination, with the Rayleigh statistical test applied for assessment of the null hypothesis based on the distribution’s critical value. Watson’s *U*^2^ goodness-of-fit test for the von Mises distribution assesses the adjustment of the empirical phase distribution to the theoretical distribution based on the mean phase and its dispersion.

## 3. Results and Discussion

**Figure 4:**
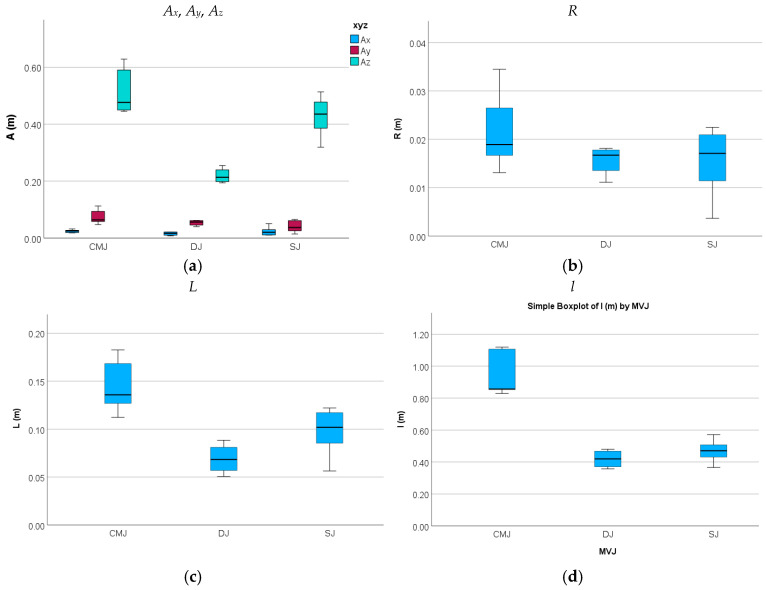
Medio-lateral *A_x_*, anteroposterior *A_y_*, and vertical *A_z_* amplitudes (**a**), mean radial distance *R* (**b**), and 2D and 3D lengths *L* and *l* (**c**,**d**) of the WB COM trajectory during the impulse phase of the CMJ, DJ, and SJ trials performed.

Whereas the medio-lateral amplitude *A_x_* of the WB COM excursion presented lower mean values, with similar magnitudes at CMJ, DJ, and SJ (p≥0.05) (Table 2 and Table 3 and Figure 4a), the anteroposterior amplitude *A_y_* of the WB COM excursion presented a higher magnitude at CMJ than at DJ (p≥0.05) and SJ (p<0.05), with a higher value at DJ than at SJ (p≥0.05) (Table 3). As regards the vertical amplitudes *A_z_* of the WB COM excursion with higher magnitudes, CMJ presented a higher value associated with the long CM in relation to SJ (p≥0.05), starting from the squat position, whereas DJ presented the lowest mean value associated with short CM, with statistically significant differences (p<0.01) (Table 2 and Table 3 and Figure 4a).

The WB COM mean radial distance *R* presented a higher mean value at CMJ than at DJ and SJ, all without statistically significant differences (p≥0.05) (Table 4 and Table 5 and Figure 4b). The 2D transversal length *L* of WB COM excursion presented a higher value at CMJ in relation to SJ, both higher than DJ, with statistically significant differences (p<0.05), and the 3D length *l* of WB COM excursion presented a higher value at CMJ in relation to SJ and DJ (p<0.01), with a lower value at DJ than at SJ (p≥0.05).

These values clearly point to lower amplitudes of WB COM excursion in the medio-lateral direction as a common characteristic between different CM conditions, with higher excursion in the anteroposterior direction for long CM compared to short CM and no CM.

**Figure 5:**
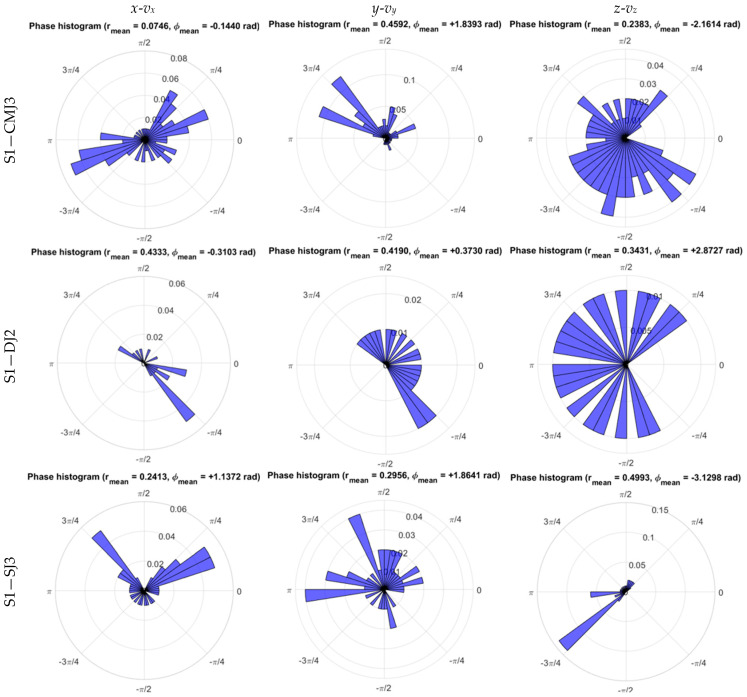
Phase histograms of medio-lateral (*x*-*v_x_*), anteroposterior (*y*-*v_y_*), and vertical (*z*-*v_z_*) phase planes of WB COM trajectory during the impulse phase of CMJ, DJ, and SJ trials performed by subject S1.

**Figure 6:**
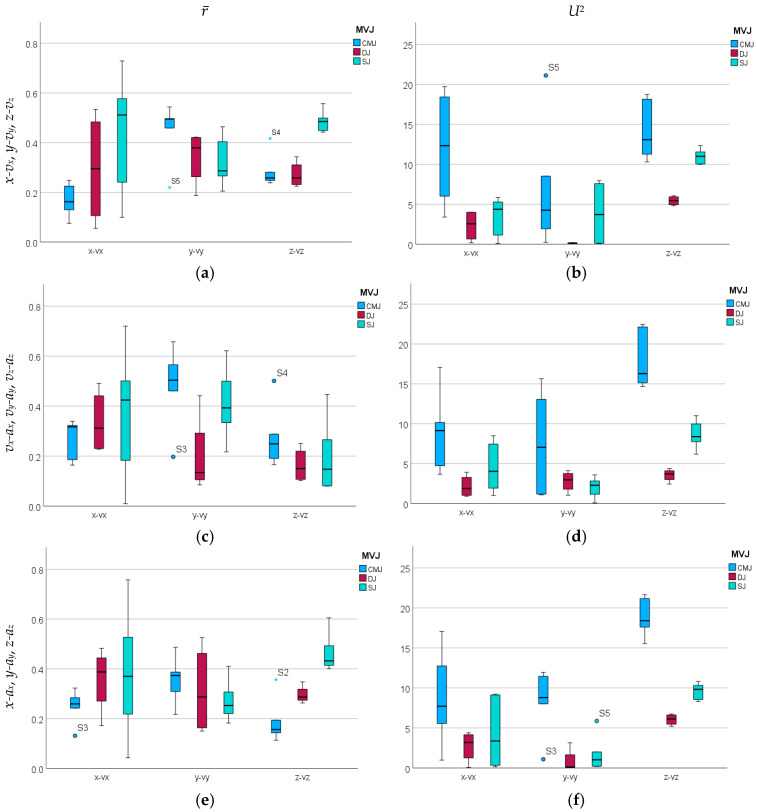
Mean resultant length $\bar{r}$ and Watson’s *U*^2^ goodness-of-fit test for the von Mises distribution of WB COM trajectory *x-v_x_*, *y-v_y_*, *z-v_z_* (**a**,**b**), *v_x_-a_x_*, *v_y_-a_y_*, *v_z_-a_z_* (**c**,**d**), and *x-a_x_*, *y-a_y_*, *z-a_z_* (**e**,**f**) phase planes during the impulse phase of the CMJ, DJ, and SJ trials performed.

WB COM presented different concentration distributions at each CM condition, as illustrated in the phase histograms of the trials performed by subject S1 at Figure 5, with CMJ showing higher $\bar{r}$ in the anteroposterior direction, and thus higher *y-v_y_* coordination, and lower $\bar{r}$ in the medio-lateral direction, with reduced *x-v_x_* coordination. In contrast, DJ presented intermediate $\bar{r}$ values, whereas SJ showed higher $\bar{r}$ at *x-v_x_* and *z-v_z_*, indicating stronger medio-lateral and vertical coordination, as seen in Figure 6a and Table 6.

The Rayleigh test of uniformity, with the null hypothesis of zero concentration for the normalized phase plane of WB COM displacement velocity, presented a higher significance at CMJ for the medio-lateral direction *x-v_x_*, with a lower percentage of rejection below or equal to 50% for ρ=0 at 5% significance, whereas the anteroposterior and vertical directions presented maximum rejection of ρ=0 with higher coordination at *y-v_y_* and *z-v_z_* (Table 7). As regards DJ, higher significance was detected in the Rayleigh test of uniformity of WB COM excursion at the medio-lateral, anteroposterior, and vertical directions, with an intermediate percentage around 50% of rejection for ρ=0 at *x-v_x_*, *y-v_y_* and no statistically significant differences from uniform distribution at *x-v_x_*, *y-v_y_*, and *z-v_z_*, indicating reduced WB COM coordination. Finally, in the medio-lateral and vertical directions, SJ presented a higher percentage (above 50%) of statistically significant differences from the uniform distribution of WB COM normalized phase, pointing to higher *x-v_x_* and *z-v_z_* coordination (Table 7).

These results emphasize the importance of the large coordination of WB COM displacement velocity in the medio-lateral direction during long CM, in contrast with intermediate coordination values during short CM on DJ and stronger coordination in the anteroposterior and vertical directions under no CM condition on SJ.

Watson’s *U*^2^ goodness-of-fit test for the von Mises distribution presented higher values at CMJ for the medio-lateral, anteroposterior, and vertical directions, whereas DJ and SJ presented higher values for the vertical direction, with higher values at CMJ than at SJ, both of which were higher than DJ (Figure 6b and Table 8). According to higher *U*^2^ values, CMJ showed maximum rejection of the von Mises hypothesis associated with the concentration around the mean phase direction of WB COM displacement velocity in the medio-lateral, anteroposterior, and vertical directions, as well as in the vertical direction on DJ and SJ (Table 8).

**Figure 7:**
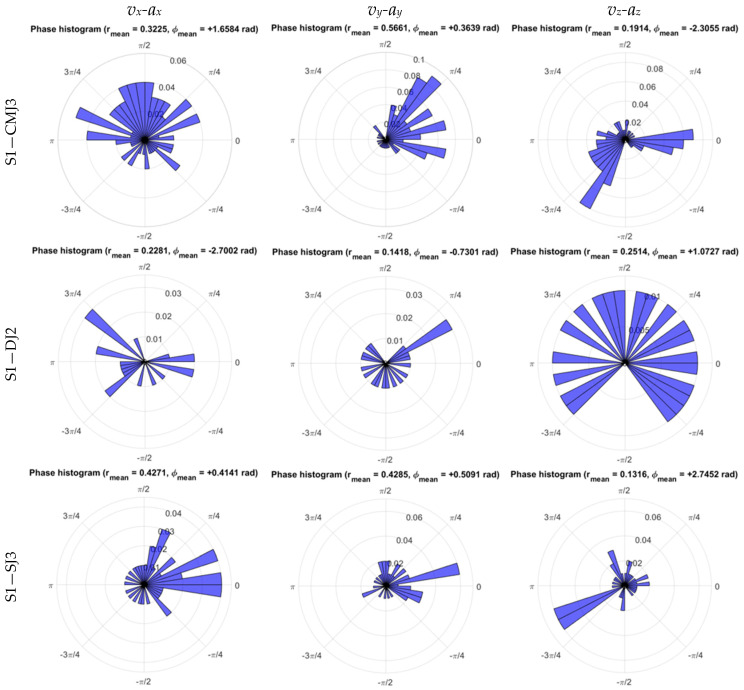
Phase histograms of medio-lateral (*v_x_-a_x_*), anteroposterior (*v_y_-a_y_*), and vertical (*v_z_-a_z_*) phase planes of WB COM trajectory during the impulse phase of the CMJ, DJ, and SJ trials performed by subject S1.

As regards the estimate of phase distribution for the velocity–acceleration of WB COM excursion during the impulse phase, illustrated in the phase histograms of trials performed by subject S1 in Figure 7, $\bar{r}$ presented higher mean value for *v_x_-a_x_* at DJ and SJ, corresponding to higher coordination in the medio-lateral direction, as well as for *v_y_-a_y_* at CMJ and SJ, corresponding to higher coordination in the anteroposterior direction, as shown in Figure 6c and Table 9.

Detected higher $\bar{r}$ mean values of the phase distribution for the velocity–acceleration of WB COM excursion highlight the differences between the impulse phases, with stronger coordination in the medio-lateral direction during short CM and no CM, whereas stronger coordination in the anteroposterior direction was detected under long and no CM conditions.

**Figure 8:**
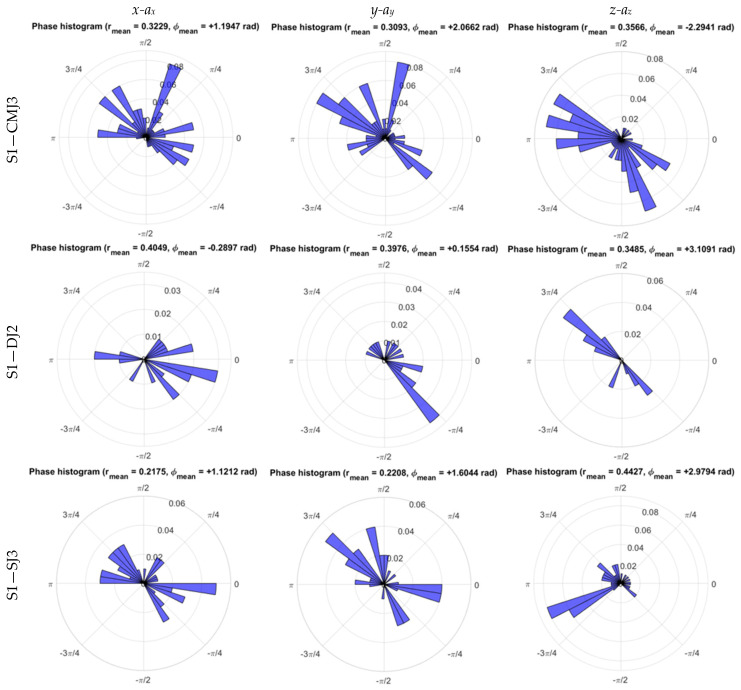
Phase histograms of medio-lateral (*x-a_x_*), anteroposterior (*y-a_y_*), and vertical (*z-a_z_*) phase planes of WB COM trajectory during the impulse phase of the CMJ, DJ, and SJ trials performed by subject S1.

The estimate of phase distribution for the displacement–acceleration of WB COM excursion during the impulse phase, illustrated in the phase histograms of trials performed by subject S1 in Figure 8, presented a higher mean $\bar{r}$ for *x-a_x_* at DJ and SJ, corresponding to higher coordination in the medio-lateral direction, whereas $\bar{r}$ for *y-a_y_* presented a higher mean at CMJ and *z-a_z_* exhibited a higher mean $\bar{r}$ at SJ, as shown in Figure 6e and Table 10.

Finally, the displacement–acceleration of the WB COM excursion led to the reinforcement of stronger coordination based on higher $\bar{r}$ mean values of the phase distribution in the medio-lateral direction for the short CM and no CM, as well as in the anteroposterior direction for the long CM condition.

The Rayleigh test of uniformity, with the null hypothesis of zero concentration for the normalized phase plane of WB COM velocity–acceleration, presented at CMJ a higher percentage of rejection for ρ=0 at 5% significance, with lower significance for the medio-lateral, anteroposterior, and vertical directions corresponding to higher coordination of *v_x_*-*ax*, *v_y_*-*a_y_*, and *v_z_*-*a_z_*. In contrast, DJ showed a reduced percentage of rejection for ρ=0 at 5% significance, whereas SJ presented a higher percentage of rejection for ρ=0 at 5% significance for the anteroposterior direction with higher coordination of *v_y_*-*a_y_* (Table 11).

Regarding the phase plane from the velocity–acceleration of WB COM in each anatomical direction, the mean radial distance and Rayleigh test rejection rate of the null hypothesis ρ=0 presented complementary roles, pointing to stronger coordination according to both metrics (Table 9 and Table 11) for the antero-posterior direction at long CM on CMJ and no CM on SJ. In contrast, a higher mean $\bar{r}$ was detected in the medio-lateral direction for short CM on DJ and no CM on SJ (Table 9), with a higher percentage of rejection for *ρ* = 0 at the Rayleigh test of uniformity on CMJ for the antero-posterior and vertical directions (Table 11).

Watson’s *U*^2^ goodness-of-fit test for the von Mises distribution presented at CMJ, DJ, and SJ generalized maximum rejection of the hypothesis associated with the concentration around the mean phase direction for *v_x_*-*a_x_*, *v_y_*-*a_y_*, and *v_z_*-*a_z_* (Table 12), indicating the rejection of the $\bar{\phi }$ as a unique descriptor of the velocity–acceleration phase distribution for WB COM excursion during the impulse phase, as well as higher *U*^2^ values at CMJ than at DJ and SJ, as plotted at Figure 6d.

The Rayleigh test of uniformity, with the null hypothesis of zero concentration for the displacement–acceleration normalized phase plane of WB COM, presented maximum rejection for ρ=0 at 5% significance, with lower significance for the medio-lateral and anteroposterior directions at CMJ and the vertical direction at SJ, corresponding to higher coordination for *x*-*a_x_* and *y-a_y_* at CMJ, as well as for *z-a_z_* at SJ impulse phases (Table 13).

As regards the phase plane from the displacement–acceleration of WB COM in each anatomical direction, the mean radial distance and Rayleigh test rejection rate of the null hypothesis ρ=0 also presented a complementary role, with concurrent metrics pointing to stronger coordination in the anteroposterior direction for CMJ and in the vertical direction for SJ, whereas stronger medio-lateral coordination was captured by the Rayleigh test at CMJ and higher mean $\bar{r}$ at DJ and SJ, as shown in Table 10 and Table 13.

Finally, concerning Watson’s *U*^2^ goodness-of-fit test for the von Mises distribution associated with the mean phase direction of WB COM displacement–acceleration, CMJ showed maximum rejection of the von Mises hypothesis associated with the concentration around mean phase in the *x-a_x_*, *y-a_y_*, and *z-a_z_* phase planes, along with DJ and SJ for *z-a_z_*, with higher *U^2^* values, as illustrated in Figure 6f and Table 14.

Maximum rejection rates of the von Mises hypothesis for the WB COM displacement–acceleration in the medio-lateral, anteroposterior, and vertical directions on CMJ and vertical direction on DJ and SJ, presented at Table 14, are also shared with the corresponding maximum rejection rates of the von Mises hypothesis for the WB COM displacement-velocity and velocity–acceleration presented in Table 8 and Table 12, whereas DJ and SJ medio-lateral and DJ anteroposterior maximum rejection rates of the von Mises hypothesis for WB COM are restricted to the velocity–acceleration phase plane (Table 12).

Watson’s *U*^2^ goodness-of-fit test for the von Mises distribution is based on the mean direction and concentration factor estimated from the phase sample. Thus, the results of this test present stronger relevance for conditions with a higher $\bar{r}$ estimate of the population mean length *ρ* of the phase distribution concentration and a higher rejection rate of the Rayleigh test of uniformity. Additionally, the relevance of the mean direction $\bar{\phi }$ is also determined by the level $\bar{r}$ of phase concentration. For this reason, primary attention was focused on $\bar{r}$ values of phase concentration rather than the mean direction $\bar{\phi }$, with illustrative phase histograms plotted for the selected subject S1.

To the best of our knowledge, this is the first study on WB COM excursion in the transversal anatomical plane during long, short, and no CM impulse phases, with previous studies predominantly focusing on the vertical direction, despite WB COM movement in the transversal direction under different CM conditions. The expansion of WB COM movement analysis from vertical to medio-lateral and anteroposterior directions during long, short, and no CM impulse phases has thus led to new results of global metrics, namely for the medio-lateral and antero-posterior amplitudes, the transversal mean radial distance, and the 2D and 3D lengths of the path during WB COM excursion under different CM conditions, with shared and specific features for each CM condition. Also, the expansion of phase plane analysis from vertical to medio-lateral and antero-posterior directions, as well as the extension of phase plane analysis to phase space analysis in each anatomical direction, has also led to new knowledge on shared and specific CM results.

The proposed workflow thus corresponds to the comparison of global metrics in the medio-lateral and anteroposterior directions, along with the transversal plane, in addition to the vertical direction metrics of WB COM excursion during long, short, and no CM, as well as the coordination assessment in the phase space of WB COM for each degree of freedom (d.o.f) concerning displacement, velocity, and acceleration in the mediolateral, anteroposterior, and vertical directions. For this, whereas mean radial distance provides valid information on coordination at each d.o.f. based on the $\bar{r}$ value of the phase concentration, the Rayleigh test contributes to its validation based on the rejection of the uniform phase distribution for the selected significance. Finally, Watson’s U^2^ goodness-of-fit test for the von Mises distribution was applied to assess the adjustment of the empirical phase distribution to the theoretical distribution based on the mean phase and its dispersion. Nevertheless, the validity of this final step depends on the results of the previous steps due to the need for phase concentration and the rejection of its uniformity to access the adjustment to the distribution based on the determined phase location and concentration.

The results obtained are consistent with the expected ones, and previous studies postulate higher neuromuscular control and increased performance of untrained subjects at long CM on CMJ compared to no CM on SJ, and short CM on DJ with the poorest performance indicated by the lowest value of the MVJ height [21]. Thus, whereas no statistical differences were detected in the medio-lateral amplitudes of WB COM excursion during impulse phases across different CM conditions, indicating the invariance of this metric with lower magnitude regardless of the type of impulse, a clear distinction was detected in the anterior-posterior direction, with higher amplitudes of WB COM excursion at long CM during the CMJ impulse phase compared to short CM on DJ and no CM on SJ, which is in agreement with the higher performance on CMJ than on DJ and SJ [21]. The higher amplitude of the vertical WB COM excursion at CMJ compared to SJ, both of which are higher than DJ, is intrinsically associated with the experimental protocol, with long CM on CMJ, the starting squat position on SJ, and short CM on DJ.

Additionally, whereas the mean radial distance *R* of WB COM to its mean presented, during the impulse phase, higher values at CMJ compared to DJ and SJ, with no statistically significant differences, the 2D length *L* of the path in the transversal plane during WB COM excursion led to the detection of statistically significant differences, with higher values at CMJ compared to SJ, both of which were higher than DJ, aligning with MVJ performance, pointing to the importance of the transversal metric of WB COM excursion during the impulse phase in relation to MVJ performance. Finally, despite the importance of the global 3D length *l* of the path during COM excursion, the inclusion of the WB COM vertical dimension leads to its dominance over the transversal dimensions, resulting in a loss of statistical differences in the 3D metric between DJ and SJ, while maintaining the differences (p<0.05) with CMJ.

As regards the phase plane metrics of WB COM, long CM at CMJ presented a higher level of coordination in the mean radial distance and Rayleigh test rejection across different phase planes for the displacement–velocity, velocity–acceleration, and displacement–acceleration, all in the antero-posterior direction, as listed in Table A1, Table A2 and Table A3 in Appendix A. The next level of coordination was observed at no CM on SJ for the displacement–velocity in the medio-lateral and vertical directions, the velocity–acceleration in the antero-posterior direction, and the displacement–acceleration in the vertical direction, and the lowest degree of coordination was found at short CM on DJ, in agreement with assessed performance [13], which showed lower MVJ height, indicating that coordination is a good performance predictor for each CM.

Also, whereas the 54 trials assessment performed by a group of six subjects can be accepted as an exploratory and proof-of-concept study, the limitations for generalizing the interpretation of the results must be considered, with the need for larger sample studies to compare coordination across different populations, tasks, and conditions.

## 4. Conclusions and Future Work

Selected metrics of transversal plane and space WB COM excursion led to the detection of shared features under long, short, and no CM jump conditions, such as lower amplitudes in the medio-lateral direction relative to the antero-posterior direction, both of which were lower than the vertical amplitude, without statistically significant differences at 5% significance for the medio-lateral amplitudes across long, short, and no CM, and higher antero-posterior and vertical amplitudes were observed at long CM impulse, with differences noted between the higher antero-posterior amplitude on short CM than at no CM p≥0.05, and the opposite for vertical amplitude p<0.05. Similarly, whereas WB COM mean radial distance presented a higher value at CMJ than at DJ and SJ, all without statistically significant differences at 5% significance, the 2D transversal and 3D space lengths of WB COM excursion showed higher values at CMJ with statistical differences, p<0.05, and higher values on SJ than DJ, with p<0.05 at the 2D metric and p≥0.05 at the 3D metric.

As regards the phase plane metrics of WB COM displacement–velocity in each anatomical direction, mean radial distance and Rayleigh test rejection rate of the null hypothesis and significance play a complementary role, enabling us to distinguish long CM, on CMJ lower coordination in the medio-lateral direction with higher coordination in the antero-posterior and vertical directions, from short CM at DJ with lower coordination in the medio-lateral, antero-posterior, and vertical directions, as well as from SJ with no CM and higher coordination in the medio-lateral and vertical directions. Additionally, Watson’s *U*^2^ goodness-of-fit test for the von Mises distribution, using the mean direction and concentration factor estimated from the sample, presented a clear distinction between CMJ with long CM and maximum rejection of the null hypothesis for the phase angle following the von Mises distribution around the displacement-velocity mean angle in the medio-lateral, antero-posterior, and vertical directions, compared to DJ with short CM, and SJ with no CM, with maximum rejection in the vertical direction. Nevertheless, this result must be looked at carefully due to the possibility of multimodal distribution of circular phase and the specificity of the mean phase in circular data. For this reason, WB COM generalized phase space analysis presents itself as a potentially interesting approach for the expansion of the performed phase plane analysis in each anatomical direction, particularly in relation to each CM condition.

WB COM generalized phase space analysis led to the detection of shared higher-level coordination on long CM impulse phases at CMJ for the antero-posterior direction at different phase plane displacement–velocity, velocity–acceleration, and displacement–acceleration, with no CM at SJ sharing higher coordination at displacement–velocity and displacement–acceleration phase planes for the vertical direction, and higher coordination solely detected on SJ for displacement–velocity in the medio-lateral and velocity–acceleration in the antero-posterior directions. Additionally, Watson’s *U*^2^ goodness-of-fit test for the von Mises distribution based on mean phase direction and concentration factor presented a higher level of maximum rejection rate at CMJ in relation to DJ and SJ, in agreement with higher MVJ height results from previous studies and suggesting future research interest in the quantification of this metric’s relation with MVJ performance at larger and different trials.

Whereas the assessed trials were performed in an aggregated manner, the higher variability between trials points to the need for future research involving segmentation and aggregated subgroup analysis based on mean phase similarity, as well as a sequential approach based on successive application of presented metrics such as mean phase and resultant length, the Rayleigh statistical test for circular data, and Watson’s *U*^2^ goodness-of-fit test for the von Mises distribution based on mean direction and concentration factor from generalized phase space from displacement–velocity, velocity–acceleration, and displacement–acceleration phase planes of WB COM excursion during long, short, and no CM and SSC on MVJ impulse.

Based on the achieved quantitative results, the complementary role of global and phase-plane selected metrics emerged, highlighting differences between the anatomical directions of the COM movement during impulse phases and CM conditions. The shared lower amplitudes of the COM excursion in the medio-lateral direction point to the independence of the CM type with respect to this metric, as well as to its reduced impact compared to COM antero-posterior excursion across different impulse phase performances. The alignment of the larger COM excursion in the antero-posterior direction during CMJ impulse phase, along with higher performance compared to SJ and DJ, points also to the importance of this metric in relation to jumping performance. Finally, the shared higher-level coordination in the antero-posterior direction for the different phase planes regarding displacement–velocity, velocity–acceleration, and displacement–acceleration during the long CM impulse phases compared to SJ and DJ reinforces the importance of phase metric analysis in its influence on motor control and jumping performance.

By providing quantitative descriptors of WB COM coordination that are sensitive to countermovement strategy, the present approach offers a foundation for future studies aiming to integrate these metrics into predictive or diagnostic models of movement performance. Such applications would facilitate the translation of the proposed dynamic system analysis into practical settings and enhance its utility for both research and applied domains. Furthermore, the generalized phase space framework introduced in this study may serve as a dynamic systems-based tool for comparing movement coordination across populations, task constraints, or fatigue conditions based on larger samples.

## Figures and Tables

**Table 1:** Long, short, and no CM of WB COM corresponding to CMJ, DJ, and SJ.

CMJ ↔ long CM	DJ ↔ short CM	SJ ↔ no CM

**Table 2:** Mean (μ) and standard deviation (σ) of the medio-lateral *A_x_*, anteroposterior *A_y_*, and vertical *A_z_* amplitudes of WB COM excursion during the impulse phase at CMJ, DJ, and SJ.

	*A_x_* (m)	*A_y_* (m)	*A_z_* (m)
CMJ	0.024 ± 0.006	0.075 ± 0.027	0.518 ± 0.085
DJ	0.016 ± 0.007	0.054 ± 0.010	0.219 ± 0.027
SJ	0.024 ± 0.015	0.040 ± 0.020	0.428 ± 0.069

**Table 3:** Mean differences ($\mu_{1}-\mu_{2}$) and significances (*p*) for the comparisons of medio-lateral *A_x_*, anteroposterior *A_y_*, and vertical *A_z_* amplitudes of WB COM excursion during CMJ, DJ, and SJ impulse phases with statistically significant differences * (p<0.05) and ** (p<0.01).

$\mu_{1}-\mu_{2}$ (*p*)	*A_x_* (m)	*A_y_* (m)	*A_z_* (m)
CMJ—DJ	0.009 (0.075)	0.021 (0.159)	0.299 ** (<10^−3^)
CMJ—SJ	<0.001 (0.934)	0.035 * (0.035)	0.090 (0.084)
DJ—SJ	−0.008 (0.349)	0.014 (0.250)	−0.209 ** (<10^−3^)

**Table 4:** Mean (μ) and standard deviation (σ) of the mean radial distance *R* and 2D and 3D length *L* and *l* of WB COM excursion during the impulse phase at CMJ, DJ, and SJ.

	*R* (m)	*L* (m)	*l* (m)
CMJ	0.022 ± 0.009	0.145 ± 0.029	0.953 ± 0.146
DJ	0.016 ± 0.003	0.069 ± 0.016	0.419 ± 0.058
SJ	0.015 ± 0.007	0.097 ± 0.025	0.470 ± 0.079

**Table 5:** Mean differences ($\mu_{1}-\mu_{2}$) and significances (*p*) for the comparisons of mean radial distance *R* and 2D and 3D length *L* and *l* of WB COM excursion during CMJ, DJ, and SJ impulse phases with statistically significant differences * (p<0.05) and ** (p<0.01).

$\mu_{1}-\mu_{2}$ (*p*)	*R* (m)	*L* (m)	*l* (m)
CMJ—DJ	0.006 (0.106)	0.076 ** (0.001)	0.534 ** (<10^−3^)
CMJ—SJ	0.007 (0.102)	0.048 ** (0.008)	0.483 ** (<10^−3^)
DJ—SJ	0.001 (0.478)	−0.028 * (0.039)	−0.051 (0.140)

**Table 6:** Mean (μ) and standard deviation (*s*) of mean radial distance ($\bar{r}$) for the medio-lateral *x-v_x_*, anteroposterior *y-v_y_*, and vertical *z-v_z_* phase planes of WB COM excursion during the impulse phase of CMJ, DJ, and SJ.

$\bar{r}$	*x-v_x_*	*y-v_y_*	*z-v_z_*
CMJ	0.168 ± 0.070	0.443 ± 0.128	0.288 ± 0.074
DJ	0.295 ± 0.226	0.342 ± 0.110	0.271 ± 0.053
SJ	0.445 ± 0.232	0.319 ± 0.096	0.486 ± 0.041

**Table 7:** Rejection rate for the Rayleigh test (*p* < 0.05) of the mean radial distance ($\bar{r}$) for the medio-lateral *x-v_x_*, anteroposterior *y-v_y_*, and vertical *z-v_z_* phase planes of WB COM excursion during the impulse phase at CMJ, DJ, and SJ.

% *Ra* (*r* ≠ 0); *p*	*x-v_x_*	*y-v_y_*	*z-v_z_*
CMJ	40%; 0.231	100%; 0.003	100%; 0.006
DJ	50%; 0.353	50%; 0.158	0%; 0.252
SJ	67%; 0.132	50%; 0.063	100%; <10^−3^

**Table 8:** Watson’s *U*^2^ goodness-of-fit test for the von Mises distribution for the medio-lateral *x-v_x_*, anteroposterior *y-v_y_*, and vertical *z-v_z_* phase planes of WB COM excursion during the impulse phase at CMJ, DJ, and SJ.

*U*^2^; % *p* < 0.05	*x-v_x_*	*y-v_y_*	*z-v_z_*
CMJ	11.97; 100%	7.20; 100%	14.31; 100%
DJ	2.32; 75%	0.13; 25%	5.43; 100%
SJ	3.52; 83%	3.85; 67%	10.98; 100%

**Table 9:** Mean (μ) and standard deviation (*s*) of mean radial distance ($\bar{r}$) for the medio-lateral *v_x_-a_x_*, anteroposterior *v_y_-a_y_*, and vertical *v_z_-a_z_* phase planes of WB COM excursion during the impulse phase at CMJ, DJ, and SJ.

$\bar{r}$	*v_x_-a_x_*	*v_y_-a_y_*	*v_z_-a_z_*
CMJ	0.266 ± 0.083	0.477 ± 0.173	0.279 ± 0.133
DJ	0.336 ± 0.129	0.199 ± 0.164	0.163 ± 0.070
SJ	0.377 ± 0.249	0.410 ± 0.141	0.195 ± 0.141

**Table 10:** Mean (μ) and standard deviation (σ) of mean radial distance ($\bar{r}$) for the medio-lateral *x-a_x_*, anteroposterior *y-a_y_*, and vertical *z-a_z_* phase planes of WB COM excursion during the impulse phase at CMJ, DJ, and SJ.

$\bar{r}$	*x-a_x_*	*y-a_y_*	*z-a_z_*
CMJ	0.248 ± 0.072	0.355 ± 0.100	0.193 ± 0.096
DJ	0.357 ± 0.133	0.312 ± 0.180	0.296 ± 0.037
SJ	0.381 ± 0.254	0.271 ± 0.082	0.463 ± 0.077

**Table 11:** Rejection rate for the Rayleigh test (*p* < 0.05) of the mean radial distance ($\bar{r}$) for the medio-lateral *v_x_-a_x_*, anteroposterior *v_y_-a_y_*, and vertical *v_z_-a_z_* phase planes of WB COM excursion during the impulse phase at CMJ, DJ, and SJ.

% *Ra* (*r* ≠ 0); *p*	*v_x_-a_x_*	*v_y_-a_y_*	*v_z_-a_z_*
CMJ	80%; 0.051	80%; 0.020	80%; 0.025
DJ	25%; 0.175	25%; 0.568	0%; 0.575
SJ	67%; 0.213	83%; 0.036	17%; 0.418

**Table 12:** Watson’s *U*^2^ goodness-of-fit test for the von Mises distribution for the medio-lateral *v_x_-a_x_*, anteroposterior *v_y_-a_y_*, and vertical *v_z_-a_z_* phase planes of WB COM excursion during the impulse phase at CMJ, DJ, and SJ.

*U*^2^; % *p* < 0.05	*v_x_-a_x_*	*v_y_-a_y_*	*v_z_-a_z_*
CMJ	8.96; 100%	7.60; 100%	18.14; 100%
DJ	2.15; 100%	2.78; 100%	3.56; 100%
SJ	4.49; 100%	2.04; 83%	8.62; 100%

**Table 13:** Rejection rate for the Rayleigh test (*p* < 0.05) of the mean radial distance ($\bar{r}$) for the medio-lateral *x-a_x_*, anteroposterior *y-a_y_*, and vertical *z-a_z_* phase planes of WB COM excursion during the impulse phase at CMJ, DJ, and SJ.

% *Ra* (*r* ≠ 0); *p*	*x-a_x_*	*y-a_y_*	*z-a_z_*
CMJ	80%; 0.077	100%; 0.006	40%; 0.166
DJ	50%; 0.157	50%; 0.298	0%; 0.160
SJ	50%; 0.189	33%; 0.107	100%; <10^−3^

**Table 14:** Watson’s *U*^2^ goodness-of-fit test for the von Mises distribution for the medio-lateral *x-a_x_*, anteroposterior *y-a_y_*, and vertical *z-a_z_* phase planes of WB COM excursion during the impulse phase at CMJ, DJ, and SJ.

*U*^2^; % *p* < 0.05	*x-a_x_*	*y-a_y_*	*z-a_z_*
CMJ	8.82; 100%	8.26; 100%	18.87; 100%
DJ	2.71; 75%	0.87; 25%	6.05; 100%
SJ	4.27; 83%	1.72; 83%	9.61; 100%

## Data Availability

The data underlying this study is available from the corresponding author upon request.

## Abbreviations

The following abbreviations are used in this manuscript:

WB	Whole-body
COM	Center of mass
MVJ	Maximum vertical jump
CM	Countermovement
GRF	Ground reaction force
COP	Center of pressure
SSC	Stretch-shortening cycle
CMJ	Countermovement jump
DJ	Drop jump
SJ	Squat jump

## Appendix A

**Table A1:** Compilation of the mean radial distance $\bar{r}$, Rayleigh *Ra*, and Watson’s *U*^2^ goodness-of-fit test for the von Mises distribution of the displacement–velocity coordination in the medio-lateral *x*-*v_x_*, anteroposterior *y*-*v_y_*, and vertical *z*-*v_z_* directions for CMJ, DJ, and SJ.

Displacement- Velocity	Medio-Lateral *x*-*v_x_*	Anteroposterior *y*-*v_y_*	Vertical *z*-*v_z_*
CMJ	$<\bar{r}$ <*x-vx* coordination	$>\bar{r}$ >*y-vy* coordination	
	>α (Ra) ρ=0 <% ρ≠0; <*x-vx* coordination	<α (Ra) ρ=0 >% ρ≠0; >*y-vy* coordination	<α (Ra) ρ=0 >% ρ≠0; >*z-vz* coordination
	$>U^{2}\sim \mathrm{Concentration}\;\bar{\phi }$ 100% Rejection *U*^2^ = 0	$>U^{2}\sim \mathrm{Concentration}\;\bar{\phi }$ 100% Rejection *U*^2^ = 0	$>U^{2}\sim \mathrm{Concentration}\;\bar{\phi }$ 100% Rejection *U*^2^ = 0
DJ	$\mathrm{Intermediate}\;\bar{r}$	$\mathrm{Intermediate}\;\bar{r}$	$\mathrm{Intermediate}\;\bar{r}$
	>α (Ra) ρ=0 <% ρ≠0; <*x-vx* coordination	>α (Ra) ρ=0 <% ρ≠0; <*y-vy* coordination	>α (Ra) ρ=0 <% ρ≠0; <*z-vz* coordination
	$<U^{2}\;\mathrm{Concentration}\;\bar{\phi }$ 75% Rejection *U*^2^ = 0	$<U^{2}\;\mathrm{Concentration}\;\bar{\phi }$ 25% Rejection *U*^2^ = 0	$>U^{2}\sim \mathrm{Concentration}\;\bar{\phi }$ 100% Rejection *U*^2^ = 0
SJ	$>\bar{r}$ >*x-vx* coordination		$>\bar{r}$ >*z-vz* coordination
	<α (Ra) ρ=0 >% ρ≠0; >*x-vx* coordination		<α (Ra) ρ=0 >% ρ≠0; >*z-vz* coordination
	$<U^{2}\;\mathrm{Concentration}\;\bar{\phi }$ 83% Rejection *U*^2^ = 0	$<U^{2}\;\mathrm{Concentration}\;\bar{\phi }$ 67% Rejection *U*^2^ = 0	$>U^{2}\sim \mathrm{Concentration}\;\bar{\phi }$ 100% Rejection *U*^2^ = 0

**Table A2:** Compilation of the mean radial distance $\bar{r}$, Rayleigh *Ra*, and Watson’s *U*^2^ goodness-of-fit test for the von Mises distribution of the velocity–acceleration coordination in the medio-lateral ***v_x_*-*a_x_***, anteroposterior ***v_y_*-*a_y_***, and vertical ***v_z_*-*a_z_*** directions for CMJ, DJ, and SJ.

Velocity- Acceleration	Medio-Lateral *v_x_*-*a_x_*	Anteroposterior *v_y_*-*a_y_*	Vertical *v_z_*-*a_z_*
CMJ		$>\bar{r}$ >*vy-ay* coordination	
	<α (Ra) ρ=0 >% ρ≠0; >*vx-ax* coordination	<α (Ra) ρ=0 >% ρ≠0; >*vy-ay* coordination	<α (Ra) ρ=0 >% ρ≠0; >*vz-az* coordination
	$>U^{2}\sim \mathrm{Concentration}\;\bar{\phi }$ 100% Rejection *U*^2^ = 0	$>U^{2}\sim \mathrm{Concentration}\;\bar{\phi }$ 100% Rejection *U*^2^ = 0	$>U^{2}\sim \mathrm{Concentration}\;\bar{\phi }$ 100% Rejection *U*^2^ = 0
DJ	$>\bar{r}$ >*vx-ax* coordination		
	>α (Ra) ρ=0 <% ρ≠0; <*vx-ax* coordination	>α (Ra) ρ=0 < % ρ≠0; <*vy-ay* coordination	>α (Ra) ρ=0 <% ρ≠0; <*vz-az* coordination
	$>U^{2}\sim \mathrm{Concentration}\;\bar{\phi }$ 100% Rejection *U*^2^ = 0	$>U^{2}\sim \mathrm{Concentration}\;\bar{\phi }$ 100% Rejection *U*^2^ = 0	$>U^{2}\sim \mathrm{Concentration}\;\bar{\phi }$ 100% Rejection *U*^2^ = 0
SJ	$>\bar{r}$ >*vx-ax* coordination	$>\bar{r}$ >*vy-ay* coordination	
		<α (Ra) ρ=0 >% ρ≠0; >*vy-ay* coordination	
	$>U^{2}\sim \mathrm{Concentration}\;\bar{\phi }$ 100% Rejection *U*^2^ = 0		$>U^{2}\sim \mathrm{Concentration}\;\bar{\phi }$ 100% Rejection *U*^2^ = 0

**Table A3:** Compilation of the mean radial distance $\bar{r}$, Rayleigh *Ra*, and Watson’s *U*^2^ goodness-of-fit test for the von Mises distribution of the displacement–acceleration coordination in the medio-lateral ***x*-*a_x_***, anteroposterior *y*-*a_y_*, and vertical *z*-*a_z_* directions for CMJ, DJ, and SJ.

Displacement- Acceleration	Medio-Lateral *x*-*a_x_*	Anteroposterior *y*-*a_y_*	Vertical *z*-*a_z_*
CMJ		$>\bar{r}$ >*y-ay* coordination	
	<α (Ra) ρ=0 >% ρ≠0; >*x-ax* coordination	<α (Ra) ρ=0 >% ρ≠0; >*y-ay* coordination	
	$>U^{2}\sim \mathrm{Concentration}\;\bar{\phi }$ 100% Rejection *U*^2^ = 0	$>U^{2}\sim \mathrm{Concentration}\;\bar{\phi }$ 100% Rejection *U*^2^ = 0	$>U^{2}\sim \mathrm{Concentration}\;\bar{\phi }$ 100% Rejection *U*^2^ = 0
DJ	$>\bar{r}$ >*x-ax* coordination		
			$>U^{2}\sim \mathrm{Concentration}\;\bar{\phi }$ 100% Rejection *U*^2^ = 0
SJ	$>\bar{r}$ >*x-ax* coordination		$>\bar{r}$ >*z-az* coordination
			<α (Ra) ρ=0 >% ρ≠0; >*z-az* coordination
			$>U^{2}\sim \mathrm{Concentration}\;\bar{\phi }$ 100% Rejection *U*^2^ = 0
